# Clustering analysis reveals different profiles associating long-term post-COVID symptoms, COVID-19 symptoms at hospital admission and previous medical co-morbidities in previously hospitalized COVID-19 survivors

**DOI:** 10.1007/s15010-022-01822-x

**Published:** 2022-04-22

**Authors:** César Fernández-de-las-Peñas, José D. Martín-Guerrero, Lidiane L. Florencio, Esperanza Navarro-Pardo, Jorge Rodríguez-Jiménez, Juan Torres-Macho, Oscar J. Pellicer-Valero

**Affiliations:** 1 Department of Physical Therapy, Occupational Therapy, Physical Medicine and Rehabilitation, Facultad de Ciencias de la Salud, Universidad Rey Juan Carlos (URJC), Avenida de Atenas s/n, Alcorcón, 28922 Madrid, Spain; 2 Intelligent Data Analysis Laboratory, Department of Electronic Engineering, ETSE (Engineering School), Universitat de València (UV), Valencia, Spain; 3 Department of Developmental and Educational Psychology, Universitat de València (UV), Valencia, Spain; 4 Department of Medicine, Universidad Complutense de Madrid (UCM), Madrid, Spain; 5 Department of Internal Medicine, Hospital Universitario Infanta Leonor-Virgen de la Torre, Madrid, Spain

**Keywords:** COVID-19, K-means clustering, Post-COVID, Long COVID, Groups

## Abstract

**Purpose:**

To identify subgroups of COVID-19 survivors exhibiting long-term post-COVID symptoms according to clinical/hospitalization data by using cluster analysis in order to foresee the illness progress and facilitate subsequent prognosis.

**Methods:**

Age, gender, height, weight, pre-existing medical comorbidities, Internal Care Unit (ICU) admission, days at hospital, and presence of COVID-19 symptoms at hospital admission were collected from hospital records in a sample of patients recovered from COVID-19 at five hospitals in Madrid (Spain). A predefined list of post-COVID symptoms was systematically assessed a mean of 8.4 months (SD 15.5) after hospital discharge. Anxiety/depressive levels and sleep quality were assessed with the Hospital Anxiety and Depression Scale and Pittsburgh Sleep Quality Index, respectively. Cluster analysis was used to identify groupings of COVID-19 patients without introducing any previous assumptions, yielding three different clusters associating post-COVID symptoms with acute COVID-19 symptoms at hospital admission.

**Results:**

Cluster 2 grouped subjects with lower prevalence of medical co-morbidities, lower number of COVID-19 symptoms at hospital admission, lower number of post-COVID symptoms, and almost no limitations with daily living activities when compared to the others. In contrast, individuals in cluster 0 and 1 exhibited higher number of pre-existing medical co-morbidities, higher number of COVID-19 symptoms at hospital admission, higher number of long-term post-COVID symptoms (particularly fatigue, dyspnea and pain), more limitations on daily living activities, higher anxiety and depressive levels, and worse sleep quality than those in cluster 2.

**Conclusions:**

The identified subgrouping may reflect different mechanisms which should be considered in therapeutic interventions.

## Introduction

Evidence suggests that symptoms associated with the severe acute respiratory syndrome coronavirus 2 (SARS-CoV-2) infection are highly variable and heterogeneous [1]. Due to this heterogeneity, prediction models for COVID-19 (e.g., models for the general population to predict the risk of being admitted to the hospital or models to support the prognosis of patients with COVID-19) have quickly entered in the literature to support medical decision; however, almost all published prediction models are poorly described [2].

The use of machine learning methods such as clustering algorithms have been increasingly used to investigate the heterogeneity of COVID-19, identifying different clinical phenotypes with similar combinations of traits. Clustering is an unsupervised learning model, meaning that no “a priori” hypotheses about patients’ prognosis need to be injected by the clinicians; therefore, results are data-driven and unbiased from potential previously proposed groupings [3]. In fact, some studies had identified clusters of symptoms associated with suffering SARS-CoV-2 infection [4] or with in-hospitality mortality [5].

There is also increasing evidence supporting the presence of post-COVID symptoms, i.e., symptoms persisting after the acute phase of infection. The prevalence of post-COVID symptoms ranges from 35 to 60% depending on the symptom and the follow-up period [6]. Identification of factors associated with the development of post-COVID symptomatology is needed for an early monitoring of patients at a higher risk, yet the number of studies is still limited [7]. Potential identified risk factors described in the literature include female sex, high symptom load, age, longer hospital stance, and high number of comorbidities; however, these findings are based on studies including samples of < 500 patients and recruited from single centers; therefore, no definitive conclusions can be drawn [6, 7]. In fact, contradictory results are consistently found in the literature in relation to these risk factors [6, 7].

Some attempts have been conducted to identify clusters of patients according to post-COVID symptoms. Huang et al. [8], in a preprint study, identified some clusters of symptoms at the acute phase of infection associated with being long-hauler, but no subgroups of patients were identified. In another preprint, Estiri et al. [9] identified different phenotypes suggesting that the presence of anosmia, dysgeusia, chest pain, or chronic fatigue were indicators of past SARS-CoV-2 infection in the preceding 6-months in young women. Similarly, Ziauddeen et al. [10] identified that post-COVID symptoms broadly clustered into two groups, a majority cluster (88.8%) mostly exhibiting cardiopulmonary symptoms, and a second minority cluster (11.2%) showing more multisystem symptoms. Davis et al. [11] were able to identify three clusters attending to the time course of post-COVID symptoms: one group presenting symptoms that are most likely to occur early at the acute phase of infection (2 weeks), another group presenting symptoms highly stable over time, and a third group with symptoms most likely to increase sharply in the first months after the infection. Previous studies have analyzed clusters of symptoms separately, that is, COVID-19 associated symptoms at the acute phase or post-COVID symptoms [8–11]. The present study aimed to identify clusters (groups) of COVID-19 survivors exhibiting long-term post-COVID symptoms based on clinical/hospitalization data by using cluster analysis.

## Methods

This multicenter study (LONG-COVID-EXP-CM) included patients hospitalized with a positive diagnosis of SARS-CoV-2 by RT-PCR technique and radiological findings during the first wave of the pandemic (March 10th–May 31st, 2020) in five public hospitals in Madrid (Spain). From the total of all hospitalized patients during that period, a sample of 400 individuals from each hospital was randomly selected. The Local Ethics Committees of all hospitals approved the study design (HCSC20/495E, HSO25112020, HUFA20/126, HUIL 092-20, HUF/EC1517). Informed consent was obtained from subjects before collecting data.

Clinical features (i.e., age, gender, height, weight, medical comorbidities), symptoms at hospital admission, and hospitalization data (e.g., days at the hospital, intensive care unit [ICU] admission) were collected from hospital records. Participants were scheduled for a telephone interview conducted by trained healthcare professionals. A predefined list of post-COVID symptoms including fatigue, dyspnea, throat pain, cough, palpitations, anosmia, ageusia, voice problems, hair loss, skin rashes, brain fog, memory loss, musculoskeletal pain, anxiety, depressive symptoms, sleep, or gastrointestinal problems was systematically asked, although participants were free of reporting any post-COVID symptom that they suffered from. The Hospital Anxiety and Depression Scale (HADS) and the Pittsburgh Sleep Quality Index (PSQI) were used to evaluate anxiety/depressive symptoms and sleep quality, since both questionnaires can be properly evaluated by telephone [12]. Both anxiety (HADS-A, 0-21 points) and depressive (HADS-D, 0–21 points) subscales were used. We considered the cut-off scores recommended for Spanish population (HADS-A ≥ 12 points; HADS-D ≥ 10 points) suggestive of anxiety and depressive symptoms, respectively [13]. The PSQI score (0–21points) evaluated the quality of sleep during the past month, where high scores (score > 8.0 points) indicate poor sleep quality [14].

Due to the similarities between the Severe Acute Respiratory Distress Syndrome (SARS) and COVID-19, we used the Functional Impairment Checklist (FIC), a disease-specific tool for evaluating functional consequences of SARS [15]. The FIC includes four items assessing physical symptoms (shortness of breath-dyspnea- at rest or at exertion, fatigue and muscle weakness) and other four items assessing limitations in occupational, leisure/social activities, basic, and instrumental activities of daily living as result of the symptoms [15]. In this study, we analyzed the presence of each item on an individual basis, and we also calculated the FIC-symptoms and FIC-disability scores by reckoning the severity of each item evaluated on four degrees (0, no; 1, mild; 2, moderate; 3, severe).

### Clustering analysis

Clustering techniques attempt to find subgroups (i.e., clusters) of patients that are similar among themselves but different from the rest. The simplest and also the most common used clustering algorithm is k-means. Given a number of clusters K, it starts by randomly distributing K centroids (i.e., prototype patients), and assigning all the patients to their closest centroid (in terms of the Euclidean distance); then, the centroids are recomputed as the mean of all patients assigned to them; this process is repeated until convergence. Instead of random initialization, K-means++ method [16] was employed. The results for 2, 3, and 4 clusters were assessed, and, although all three tests yielded similar results, finally only the 3-cluster model is presented here for the sake of brevity. Python library scikit-learn was employed to perform the clustering [17].

### Statistical analysis of the clusters

After applying the clustering algorithm, the mean and standard deviation was computed for each feature on each cluster, and a one-way ANOVA test (Holmes–Bonferroni-corrected for multiple comparisons) was employed to find which variables had a statistically different mean value between (at least two of those) the clusters. ANOVA was calculated using Python library Scipy (ver. 1.6.2) [18] and the p-values were corrected with Python library statsmodels (ver. 0.12.1) [19].

## Results

A total of 2,000 participants were randomly selected from the involved hospitals and invited to participate. Nine refused to participate, five were not able to be contacted after three attempts, and 17 had deceased after hospital discharge. A total of 1,969 (mean age: 61, SD: 16 years, 46.4% women) were included. Participants were assessed a mean of 8.4 months (SD 1.5, range 6 to 10 months) after hospital discharge.

**Fig. 1:**
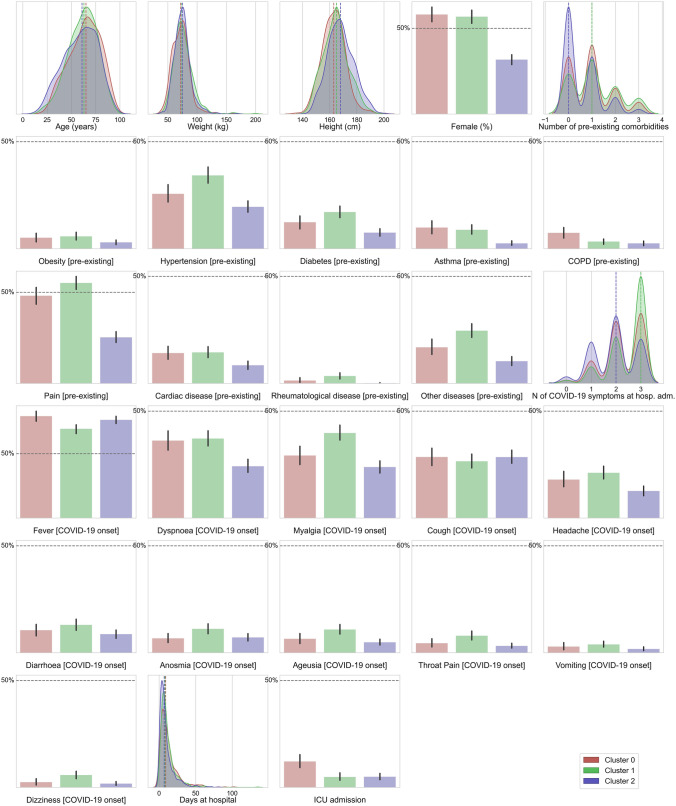
Plots of the distribution of clinical and hospitalization data for each of the three clusters. Categorical features have been represented as bar plots

**Fig. 2:**
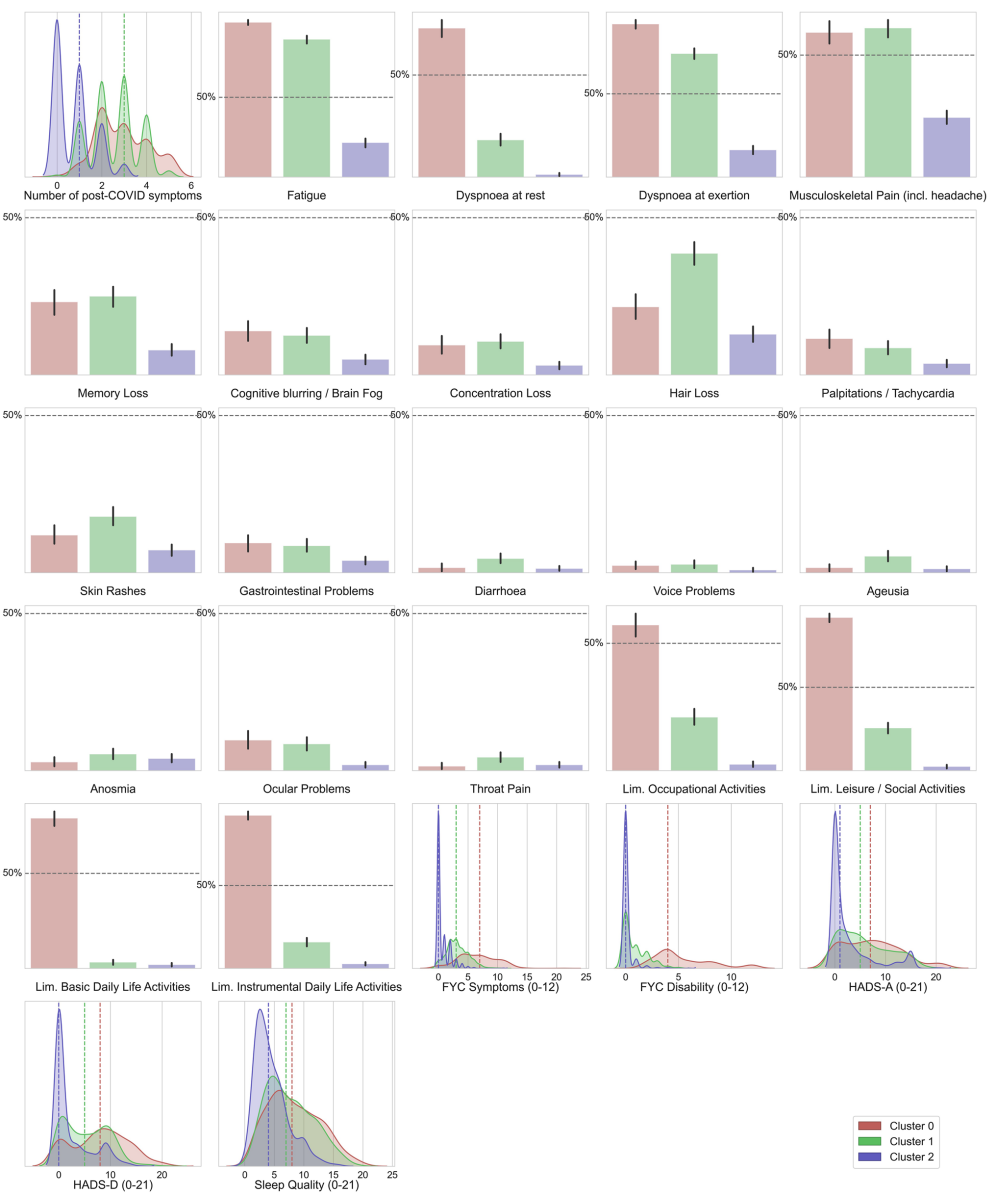
Plots of the distribution of post-COVID data for each of the three clusters. Categorical features have been represented as bar plots

Three clusters with different distributions in the variables were clearly identified, as visualized in Fig. 1 (clinical and hospitalization data) and Fig. 2 (post-COVID data**)**. Table 1 summarizes clinical and hospitalization data whereas Table 2 shows post-COVID symptoms (also mood disorders) and functional limitations for every cluster. By analyzing Table 1, we can observe that one cluster (number 2) grouped those patients with lower prevalence of medical co-morbidities and lower number of COVID-19 symptoms at hospital admission when compared with the other two (cluster 0–1), particularly, the presence of respiratory symptoms such as dyspnea, cough, and throat pain at the acute phase of infection. Cluster 2 also grouped more males and slightly younger than the other two. Clusters 0 and 1 were not significantly different neither in the prevalence of medical co-morbidities nor in COVID-19 symptoms at hospital admission.

**Table 1:** Clinical and hospitalization data according to each cluster

	Cluster 0 (*n* = 432)	Cluster 1 (*n* = 696)	Cluster 2 (*n* = 841)	*P*-value
Age (years)	63.9 ± 16.1	61.6 ± 14.9	59.3 ± 17.2	< 0.001
Weight (kg)	72.2 ± 14.6	76.2 ± 16.7	75.1 ± 13.8	< 0.001
Height (cm)	163.0 ± 8.7	164.4 ± 9.2	168.1 ± 9.5	< 0.001
Female	248 (57.4%)	403 (57.9%)	264 (31.4%)	< 0.001
Number of pre-existing co-morbidities	0.95 ± 0.9	1.15 ± 0.95	0.6 ± 0.75	< 0.001
Obesity [pre-existing]	22 (5.1%)	40 (5.75%)	26 (3.1%)	0.195
Hypertension [pre-existing]	112 (25.9%)	235 (33.75%)	167 (19.9%)	< 0.001
Diabetes [pre-existing]	54 (12.5%)	118 (16.95%)	64 (7.6%)	< 0.001
Asthma [pre-existing]	42 (9.7%)	63 (9.05%)	21 (2.5%)	< 0.001
COPD [pre-existing]	33 (7.6%)	23 (3.3%)	21 (2.5%)	< 0.001
Pain [pre-existing]	210 (48.6%)	383 (55.0%)	213 (25.3%)	< 0.001
Cardiac disease [pre-existing]	63 (14.6%)	100 (14.4%)	71 (8.4%)	0.006
Rheumatological disease [pre-existing]	6 (1.4%)	24 (3.45%)	1 (0.1%)	< 0.001
Other diseases [pre-existing]	73 (16.9%)	172 (24.7%)	87 (10.35%)	< 0.001
Number of COVID-19 symptoms at hospital admission	2.25 ± 0.8	2.5 ± 0.75	1.95 ± 0.85	< 0.001
Fever [COVID-19 onset]	347 (80.3%)	479 (68.8%)	643 (76.45%)	0.201
Dyspnea [COVID-19 onset]	156 (36.1%)	263 (37.8%)	201 (23.9%)	< 0.001
Myalgias [COVID-19 onset]	127 (29.4%)	278 (39.9%)	199 (23.7%)	< 0.001
Cough [COVID-19 onset]	125 (28.9%)	183 (26.3%)	241 (28.7%)	0.611
Headache [COVID-19 onset]	75 (17.4%)	153 (22.0%)	104 (12.4%)	< 0.001
Diarrhea [COVID-19 onset]	43 (9.9%)	94 (13.5%)	73 (8.8%)	0.082
Anosmia [COVID-19 onset]	26 (6.0%)	80 (11.5%)	61 (7.25%)	0.02
Ageusia [COVID-19 onset]	28 (6.5%)	75 (10.8%)	42 (5.0%)	0.002
Throat pain [COVID-19 onset]	20 (4.6%)	56 (8.05%)	26 (3.1%)	0.001
Vomiting [COVID-19 onset]	13 (3.0%)	27 (3.9%)	15 (1.8%)	0.195
Dizziness [COVID-19 onset]	11 (2.55%)	40 (5.75%)	15 (1.8%)	< 0.001
Days at hospital	13.0 ± 13.2	12.0 ± 11.3	9.7 ± 10.2	< 0.001
ICU admission	54 (12.5%)	34 (4.9%)	42 (5.0%)	< 0.001

COPD, chronic obstructive pulmonary disease; ICU, intensive care unit

**Table 2:** Post-COVID symptoms and functional limitations according to each cluster

	Cluster 0 (*n* = 432)	Cluster 1 (*n* = 696)	Cluster 2 (*n* = 841)	*P*-value
Number of post-COVID symptoms	2.9 ± 1.15	2.6 ± 1.05	0.75 ± 0.85	< 0.001
Fatigue	420 (97.2%)	599 (86.1%)	187 (22.25%)	< 0.001
Dyspnea at rest	317 (73.4%)	131 (18.8%)	11 (1.3%)	< 0.001
Dyspnea at exertion	398 (92.1%)	513 (73.7%)	143 (17.0%)	< 0.001
Musculoskeletal pain (including headache)	255 (59.0%)	429 (61.6%)	203 (24.15%)	< 0.001
Memory loss	99 (22.9%)	176 (25.3%)	66 (7.85%)	< 0.001
Cognitive Blurring-frain fog	62 (14.35%)	87 (12.5%)	40 (4.75%)	< 0.001
Concentration loss	42 (9.7%)	73 (10.5%)	25 (3.0%)	< 0.001
Hair loss	90 (20.8%)	274 (39.4%)	106 (12.6%)	< 0.001
Palpitations-tachycardia	50 (11.6%)	59 (8.5%)	31 (3.7%)	< 0.001
Skin rashes	53 (12.3%)	123 (17.7%)	60 (7.1%)	< 0.001
Gastrointestinal Problems	41 (9.5%)	59 (8.5%)	33 (3.9%)	0.002
Diarrhea	7 (1.6%)	31 (4.45%)	11 (1.3%)	0.002
Voice Problems	6 (1.4%)	23 (3.3%)	6 (0.7%)	0.006
Ageusia	7 (1.6%)	36 (5.2%)	10 (1.2%)	< 0.001
Anosmia	12 (2.8%)	36 (5.2%)	32 (3.8%)	0.270
Ocular problems	41 (9.5%)	60 (8.6%)	15 (1.8%)	< 0.001
Throat pain	6 (1.4%)	29 (4.2%)	15 (1.8%)	0.023
Limitation occupational Activities	244 (56.5%)	153 (22.0%)	21 (2.5%)	< 0.001
Limitation leisure/social activities	398 (92.1%)	186 (26.7%)	20 (2.4%)	< 0.001
Limitation basic daily life activities	351 (81.25%)	22 (3.15%)	16 (1.9%)	< 0.001
Limitation instrumental daily life activities	403 (93.3%)	116 (16.7%)	23 (2.7%)	< 0.001
FIC symptoms (0–12)	6.6 ± 3.25	3.25 ± 1.85	0.7 ± 1.2	< 0.001
FIC disability (0–12)	5.4 ± 2.7	0.9 ± 1.2	0.1 ± 0.5	< 0.001
HADS-A (0–21)	7.35 ± 5.7	5.9 ± 4.9	2.75 ± 4.45	< 0.001
HADS-D (0–21)	7.95 ± 5.35	5.45 ± 4.3	2.4 ± 3.65	< 0.001
Sleep quality (0–21)	8.35 ± 4.35	7.7 ± 3.9	4.55 ± 2.85	< 0.001

FIC, functional impairment checklist; HADS, Hospital Anxiety and Depression Scale (A: anxiety; D: depression)

Table 2 reveals that clusters 0 and 1 grouped individuals with higher number of long-term post-COVID symptoms (particularly respiratory post-COVID symptoms e.g., fatigue or dyspnea but also pain symptoms), more limitations on daily living activities (occupational, leisure/social, basic or instrumental), higher anxiety and depressive levels, and worse sleep quality when compared with cluster 2. Cluster 0 grouped individuals with the highest number of post-COVID symptoms and the most functionally limited, since individuals within cluster 1 exhibited less limitations with daily living activities than those in cluster 0. No significant differences in mood disorders and sleep quality were seen between clusters 0 and 1. Cluster 2 grouped the least affected patients with the lowest number of post-COVID symptoms and almost no limitations with daily living activities.

## Discussion

This research shows data from the first study of phenotypic clusters of COVID-19 survivors including COVID-19 associated symptoms at the acute phase of infection (hospital admission), long-term post-COVID symptoms, and functional limitations. We were able to identify three clusters (groups) of COVID-19 survivors: one cluster grouping patients with less affectation at hospital admission (lower number of pre-existing medical comorbidities and lower number of COVID-19 symptoms at the acute phase) and a smaller number of post-COVID symptoms with no functional limitations; two clusters (0 and 1) grouping individuals more affected at hospital admission (greater number of pre-existing comorbidities and more COVID-19 symptoms at the acute phase), a greater number of post-COVID symptoms, more limitations with daily living activities, higher levels of anxiety/depression, and worse sleep quality. Importantly, one cluster grouped those patients with more respiratory post-COVID symptoms and worse functional limitations with daily living activities (cluster 0).

Our study identified three cluster phenotypes in a population of previously hospitalized COVID-19 survivors associating previous medical co-morbidities, COVID-19 symptoms at hospital admission, long-term post-COVID symptoms, and functional repercussions. Cluster analysis grouped individuals according to the number of pre-existing medical comorbidities, the number of COVID-19 associated-symptoms at the acute phase and the number of post-COVID symptoms in the same cluster. This clustering would support the assumptions that a higher symptom load (more symptoms) at the acute phase of SARS-CoV-2 infection and a greater number of pre-existing medical comorbidities are associated with a greater likelihood of post-COVID symptoms, particularly fatigue or dyspnea, 3–6 months after infection [7]. Seeßle et al. [20] have recently observed that the number of COVID-19 associated acute symptoms was also correlated with post-COVID symptoms at 12-months follow-up. Additionally, these factors also agree with recent studies suggesting that post-COVID symptoms are more prevalent in COVID-19 patients reporting severe symptoms at onset (higher symptom load) [21] and severe-to-critical illness (those with higher medical comorbidities) at hospital [22]. Nevertheless, it should be considered that contradictory results in relation to the association between COVID-19 associated onset symptoms and post-COVID symptomatology are found in current literature [6, 7]. Additionally, it is also possible that the presence of pre-existing medical co-morbidities before the infection could contribute to development of post-COVID symptoms; however, preliminary evidence suggests that this association is specific-disease since pre-existing hypertension is associated with a higher number of post-COVID symptoms [23]; whereas diabetes [24], and asthma [25] did not.

An important finding was that one subgroup (cluster 0) exhibited more functional limitations and also more psychological stress than the other group (cluster 1) showing similar number of post-COVID symptoms. Interestingly, the most affected cluster grouped individuals (72%) reporting dyspnea as long-term post-COVID symptom, explaining why these subjects also showed more limitation during daily living activities. In fact, the presence of dyspnea at rest could also be associated with a higher presence of anxiety/depressive symptoms and a poor sleep quality due to a continuous sensation of breathlessness. Since these clusters share common characteristics and sometimes it can be difficult to recognize which cluster a patient belongs to, the development of dyspnea at rest as a post-COVID symptom, could be used for monitoring this subgroup of patients.

Similarly, our analysis also revealed a greater proportion of females in those clusters showing more long-term post-COVID symptoms (clusters 0–1). The topic of female gender as a risk factor for developing post-COVID symptoms is controversial since it is supported by some studies [26, 27] but not by others [28, 29]. Our cluster analysis grouped a higher proportion of females in those groups exhibiting more post-COVID symptoms, supporting that female gender might be a risk factor for long-term COVID symptomatology.

We believe that identification of different clusters may be of great help to clinicians to identify those cases at a higher risk of developing better or worse long-term conditions, thus directing more individualized therapeutic strategies. For instance, previous studies have associated COVID-19 onset symptoms at the acute phase with worse prognosis and higher in-hospital mortality [4, 5]. Our cluster analysis suggests that early identification of patients with a higher symptom load (a greater number of symptoms) at onset could lead to a more individualized symptomatic treatment at hospital admission. Similarly, identification of risk factors associated with the development of dyspnea as a post-COVID symptom could also improve the prognosis of individuals within the most affected group of COVID-19 survivors.

Although, to the best of our knowledge, this is the largest multicenter study investigating a classification system including COVID-19 associated onset symptoms and post-COVID symptoms using cluster analysis, some limitations should be recognized. First, we included hospitalized COVID-19 survivors; therefore, these data should not be extrapolated to non-hospitalized patients. Second, we just included Caucasian participants, extrapolation of current findings to other ethnicities should not be performed. Third, we collected post-COVID symptoms systematically in a predefined list; the first validated and reliable instrument for monitoring symptoms and impact of post-COVID symptoms (Long COVID Symptom and Impact Tools) was recently developed [30]. Finally, defining a true phenotype requires similar clinical and physiological characteristics, underlying pathobiology with identifiable biomarkers, and predictable responses to therapy. Accordingly, it would be necessary to phenotyping each of the identified clusters for a better understanding of their differences.

## Conclusions

The application of cluster analysis has identified three cluster of previous hospitalized COVID-19 survivors: one group of patients with lower number of medical comorbidities, lower number of COVID-19 symptoms at the acute phase, lower number of post-COVID symptoms and no functional limitations; and two groups of patients with greater number of medical comorbidities, more COVID-19 symptoms at the acute phase, greater number of post-COVID symptoms, and more limitations with daily living activities. This subgrouping may reflect different mechanisms which should be considered in therapeutic interventions.
